# Stigma, Social Support, and Decision Satisfaction in Terminations of Pregnancy for Medical Reasons

**DOI:** 10.1089/whr.2022.0092

**Published:** 2023-05-30

**Authors:** Tayler Hendrix, Julia Roncoroni, Brigid Magdamo, Salina Whitaker, Kornelia Zareba, Noelle Grieco

**Affiliations:** ^1^ Department of Counseling Psychology, Morgridge College of Education, University of Denver, Denver, Colorado, USA.; ^2^ Department of Obstetrics & Gynecology, College of Medicine & Health Sciences (CMHS), United Arab Emirates University (UAEU), Al Ain, UAE.; ^3^ School of Professional Psychology, University of Denver, Denver, Colorado, USA.

**Keywords:** abortion stigma, decision satisfaction, pregnancy termination, prenatal diagnosis, social support

## Abstract

**Objective:**

Existing abortion stigma research has rarely isolated the reason for termination; thus, the consequences of termination for medical reasons (TFMR) are poorly understood. We aimed to understand the association of stigma and social support with decision satisfaction in TFMR.

**Methods:**

We performed a cross-sectional study on the experiences of 132 individuals who had a TFMR in the second or third trimester. We recruited participants *via* Facebook. Most participants were non-Hispanic White (85.6%), between 31 and 40 years old (72.7%), highly educated (84.1% with a 4-year degree), and married (89.4%). Participants completed an online demographic data questionnaire, including questions about stigma and social support, and an adapted satisfaction with decision survey. We used *t*-tests to explore the connection of stigma and social support with decision satisfaction.

**Results:**

Results did not reveal an association between stigma and decision satisfaction, but showed that higher social support is associated with higher decision satisfaction. Decision satisfaction was higher in participants who experienced more than one source of support [*t*(130) = 2.527, *p* = 0.01], compared with those reporting only one source of support, and in those who experienced support from a relative [*t*(130) = 1.983, *p* = 0.049] and physician [*t*(130) = 2.357, *p* = 0.020] than in those who did not.

**Discussion:**

Social support can alleviate the suffering related to TFMR. Exploring how different forms of social support, including therapy groups, can impact decision satisfaction might help develop interventions to improve postabortion outcomes.

**Practice Implications:**

Provider training must encourage providers to (1) support patients having a TFMR and (2) connect patients with other sources of support.

## Introduction

In June 2022, the US Supreme Court decided to overturn Roe v. Wade, ending the constitutional right to have an abortion in the United States. This decision has exacerbated an already existing climate of politicization of abortion and brought forward the challenges to upholding abortion as a basic health and health care issue.^1,2^ The criminalization of abortion will likely increase stigma for individuals who terminate their pregnancies and also force them to experience constant fear of legal repercussions.^1^ Increased understanding around abortion stigma can support the development of strategies to tackle it and improve the health and well-being of those who are affected by stigma.^3^

Abortion is one of the most common medical procedures performed in the United States, with ∼25% of women reporting having had an abortion by the age of 45.^4,5^ In the United States, the number of legal abortions per year ranges between 629,898^6^ and 862,320,^7^ depending on the reporting source. Some of these abortions are terminations for medical reasons (TFMRs), which take place when (1) a fetus is considered not viable or has a poor health prognosis (detected *in utero via* prenatal screening and subsequent diagnostic tests) or (2) a mother's life or health is at risk if she continues the pregnancy.

The prevalence of TFMR is not clear in the literature; an approximation regarding the average number of abortions that are TFMRs cannot currently be provided due to many of these procedures happening in emergency medical settings, as opposed to more closely assessed abortion centers.^8,9^ Yet, major congenital fetal abnormalities occur in 3%–4% of pregnancies and are typically detected in the second and third trimesters.

The majority of pregnancies where a major congenital fetal abnormality is detected end in TFMR.^10–15^ Depending on the severity of the abnormality, this percentage can range from ∼70% to 95%.^16^ In addition, about 4% of abortions in the United States are due to maternal life or health being at risk.^17^

Despite abortion being a shared experience for many individuals and families, abortions are often concealed and considered a taboo topic of discussion.^18,19^ The stigma related to abortion often leads to underreporting.^20^ Abortion stigma occurs when negative or demeaning characteristics are attributed to those who pursue termination of pregnancy. Furthermore, the characteristics that are attributed to individuals who terminate suggest a sense of inferiority in the social context of womanhood.^3,10^

Kumar et al.^10^ posit that individuals who have abortions act in opposition to societal expectations and outdated norms set for women, such as the notion that women should engage in sex solely for the purpose of having children or the idea that women must inherently become mothers during their lifetime.^10^ Several, large, pre-Dobbs US-based studies have shown that most people who have had an abortion experienced stigma and feel the need to hide the experience from friends and family.^11,12,21^

The decision to terminate a pregnancy is one of the most difficult and emotionally demanding experiences a woman and/or a family can face in their lifetime.^22,23^ This process is further complicated by the negative effects of abortion stigma.^19,24^ While abortion itself is not linked to poor psychological outcomes,^11,25,26^ in research on individuals who have had abortions, without isolating by reason for termination, abortion stigma has been linked to poor psychological outcomes (*e.g.*, depression, prolonged grief).^19,27^

A 2022 study by Kerns et al.^11^ explored the association of perceived stigma with mental health outcomes, including satisfaction with decision, among individuals who had a TFMR for fetal anomalies in the second trimester. The study concluded that perceived social condemnation does not predict psychological outcomes.^11^ However, this study did not explore whether stigma is directly related to abortion decision satisfaction or the role of social support after the TFMR. Additionally, Kerns et al.^11^ demonstrated a predictive relationship between abortion stigma and perinatal grief, which has the potential to decrease an individual's satisfaction with their decision to pursue a TFMR.

Satisfaction with decision refers to patient satisfaction with their decisions at times when a choice has to be made and this choice should be based on critical evaluation of existing medical evidence and on patient values for outcomes.^28^ Public policy and practice are often based on the false notion that people are unsure of their decision to have an abortion and that regret of the decision to have an abortion is common.^29^

Yet, a longitudinal study of a cohort of women seeking abortions between 2008 and 2010 at 30 facilities across the United States showed that negative emotions regarding the decision to have an abortion generally decline over time.^29,30^ This study also showed that higher perceived community abortion stigma and lower social support were linked to more negative emotions such as regret, anger, guilt, and sadness about the decision to have an abortion.^30^ Yet, this study did not isolate the reason for termination.

Perceived social support can act as a buffer against the adverse impact of stigma.^31–33^ Social support has been conceptualized as “reserve capacity” for responding to stressors^34,35^ such as a TFMR and abortion stigma. In their taxonomy of resources that enable a person to withstand long-term and relatively high levels of chronic stress, Dunkel et al.^36^ included social integration/connectedness and perceived social support. Current longitudinal research highlights that social support such as shared storytelling among those who have experienced an abortion can lessen the negative effects of abortion stigma.^37^

Moreover, results from the Turnaway Study—a 5-year study into the impact of obtaining or being denied an abortion among about 1,000 women—showed that among the people who were most vulnerable to psychological distress and negative emotions, were those who lacked social support or perceived that they had been stigmatized.^2,21,26,30,38^ However, these results did not isolate individuals who terminated for medical reasons. Given that TFMRs more commonly take place in the second or third trimester when pregnancies are more public, abortion stigma may play a bigger role.^11,39,40^

The overturn of Roe v. Wade in June 2022 has exacerbated a climate where, despite the legality of TFMR in many states, individuals who have had a TFMR (and their health care providers) may experience serious abortion stigma both inside and outside the clinical setting.^24^ TFMRs often happen in the second or third trimester, falling trap to the virulent politicization of late-term abortions.^41,42^

Given that an increased understanding of experiences and biopsychosocial outcomes of individuals who have had a TFMR could lead to more successful interventions to improve wellness and mental health,^11^ our study focuses on understanding how stigma and social support relate to decision satisfaction among individuals who terminated their pregnancies for medical reasons in the second or third trimester.

## Methods

### Participants

Inclusion criteria for prospective participants included the following: (1) must be ≥18 years old; (2) must have terminated a pregnancy in the second/third trimester in the past 5 years; (3) must live in the United States both currently and at the time of termination; and (4) must be able to complete a survey in English at a fifth-grade reading level.

### Procedure

This study received approval from the University of Denver Institutional Review Board (IRB) Office. The procedures used in this study adhere to the tenets of the Declaration of Helsinki. Participants were recruited for this study *via* two Facebook groups that support families who have ended wanted pregnancies for medical reasons, “Ending a Wanted Pregnancy” and “TFMR Support Circle.” Administrators of the groups, who are parents who have gone through a TFMR, contributed to the design and implementation of this study. Their input included determining areas where we should focus the survey, how to administer/post the survey on Facebook more successfully, endorsing our study as group administrators once the survey was posted in the Facebook groups, interpreting results, and suggesting potential avenues to disseminate the infographics that we produced with study results.

At the time our study was conducted, “Ending a Wanted Pregnancy” had 2.2K members and “TFMR Support Circle” had 331 members. These groups are not visible to the public and require application-approved membership to access any of the posted information. Members from these two Facebook groups were invited to participate through a flyer shared on both groups' pages.

The flyer explained (1) the study's inclusion criteria, (2) the study protocol (either survey or survey plus an individual interview, as per participants' preference), and (3) the intended use of results for policy making and provider training.

Finally, the flyer included a link to the Qualtrics consent form and survey. Some participants filled out only the Qualtrics survey, of which portions are reported in this article. Some participants completed the survey and also virtual qualitative interviews (reported in a separate article). The present study's informed consent form and assessment battery took ∼45 minutes to complete.

In health research, Facebook offers several benefits as a recruiting platform, including the following: (1) lower costs, (2) rapid recruitment, (3) good representation of target population, and (4) better participant selection in complex demographics.^2,28^ The primary disadvantage of utilizing Facebook for recruitment is that its inclusion of participants is limited to those with internet access, as is the case in our study; this leads to an overrepresentation of White individuals.^43^

When compared with other social media platforms that have been utilized in health research (*e.g.*, Twitter and Instagram), Facebook has yielded higher rates of overall participant recruitment and also allowed recruitment materials to reach a more diverse group of potential participants; demographic data analyses of these three platforms also suggest that when compared with Facebook, Instagram and Twitter tend to reach individuals with both higher educational and socioeconomic statuses.^44,45^

Thus, to increase generalizability of results, Facebook was utilized to recruit participants for this study.

### Ethical considerations

Reflecting on the experience of the TFMR may have been triggering for some participants. Participants were provided with information on free national hotlines they can access for support, including Exhale After Abortion Text line, Connect & Breathe, and Empty Arms Bereavement Support, in the informed consent form. Participants were also encouraged to use the Facebook support groups where they found the study, “Ending a Wanted Pregnancy” and “TFMR Support Circle,” should they become uncomfortable or feel distressed during the research process.

Finally, participants were reminded during the study that their participation in this study was voluntary and that they could withdraw at any time. At the end of the study, within Qualtrics, a grounding meditation was included for participants to use as needed. This meditation was led by one of the PIs of this study (Dr. Roncoroni) who is a licensed psychologist and has led this type of meditation many times with patients and the broader community.

This grounding meditation was available for participants to regulate their emotions, focus their mind and energy on the present moment, and transition back to their daily activities.

### Measures

In this study, we describe results from the following instruments: (1) the Demographic Data Questionnaire and (2) the Satisfaction with Decision Scale (SDS).^28^

#### Demographic Data Questionnaire

This 53-question instrument was created by the research team to gather some demographic information on participants.

The questionnaire included questions about participants' (a) background (15 questions), including race/ethnicity, age, relationship status, political leaning (conservative republican, moderate/republican leaner, moderate/democrat leaner, liberal democrat, or other), religious affiliation, socioeconomic status, and place of residence at the time of the study and termination; (b) general medical information (5 questions); (c) pregnancy history (17 questions); (d) social context and social support (9 questions); (e) stigma (2 questions); and (f) beliefs regarding abortion before and after the TFMR (2 questions).

#### The Satisfaction with Decision Scale

The SDS measured patient satisfaction with health care decisions with high reliability (Cronbach's *α* = 0.89). Previous research on satisfaction with medical decisions has also supported this scale's strong reliability (Cronbach's *α* = 0.86).^28^

The survey consists of six items and includes statements such as “I am satisfied that I was adequately informed about the issues important to my decision” and “I am satisfied that this was my decision to make.” Items are scored on a 5-point Likert scale (1 = *strongly disagree*; 5 = *strongly agree*) and totaled to derive a final score, ranging from 5 to 30. Following existing literature,^39^ we modified the wording of items on the SDS to fit the experiences of TFMR patients.

### Statistical analyses

Only data from respondents who had completed 75% or more of the survey were kept and analyzed. First, descriptive statistics for both continuous and categorical variables were calculated. Second, *t*-tests were used to examine differences in the variables of interest (*i.e.*, stigma, social support, and decision satisfaction) between demographic groups.

Finally, *t-*tests were conducted to further explore the connection between the independent variables (stigma and social support) and the dependent variable (decision satisfaction) being examined in this study. Statistical analyses were conducted using SPSS, v.25.

## Results

### Demographic and descriptive information

Overall, 190 participants completed at least part of the survey; however, only those who completed all three measures used in this study were included in our study, resulting in a final sample size of 132 participants. The majority of participants in this study identified as non-Hispanic White (85.6%), while 13.6% identified as Hispanic/Latina. The majority of participants were between 31 and 40 years old (72.7%).

Over one-third of participants reported having a 4-year college/university degree and almost half reported having a graduate or professional degree. The participants primarily identified as middle or upper middle class (87.1%), married (89.4%), and full-time employed (75%). The overwhelming majority reported having private insurance (90.2%). Participants generally identified as middle class (43.9%) or upper middle class (43.2%). See Table 1 for additional demographic information.

**Table 1.** Participants' Demographic Information

	*N* (%)
Race (multiple answers allowed)	
Hispanic/Latina	18 (13.6)
American Indian/Native	1 (0.8)
Asian/Asian American	3 (2.3)
Black/African American	1 (0.8)
Caucasian/White	130 (98.5)
Native Hawaiian/other Pacific Islander	1 (0.8)
Age	
20–25 Years	2 (1.5)
26–30 Years	17 (12.9)
31–35 Years	49 (37.1)
36–40 Years	47 (35.6)
41 or older	17 (12.9)
Education	
High school graduate/GED	1 (0.8)
Trade/Tech/vocation	2 (1.5)
Some college, no degree	12 (9.1)
2-Year college	6 (4.5)
4-Year college	50 (37.9)
Profession/graduate school	61 (46.2)

	*N* (%)
Employment status	
Full-time employed	104 (78.8)
Part-time employed	10 (7.6)
Out of work/looking for work	2 (1.5)
Out of work/not looking for work	16 (12.1)
Social class (self-described)	
Lower class	2 (1.5)
Working class	8 (6.1)
Middle class	58 (43.9)
Upper middle class	57 (43.2)
Upper class	7 (5.3)
Relationship status	
Single	4 (3.0)
Married	118 (89.4)
Living with partner, not married	10 (7.6)
Insurance status	
Private insurance	119 (90.2)
Public insurance (*e.g.*, Medicare, Medicaid)	9 (6.8)
Other	4 (3.0)

Participants lived in the following regions of the United States at the time of their TFMR: West (*n* = 38; 28.8%), South (*n* = 29; 22.0%), Midwest (*n* = 30; 22.7%), and Northeast (*n* = 35; 26.5%). Of the participants, 68.1% lived in blue states (*i.e.*, US states whose voters predominantly vote for the Democratic Party); 29.9% lived in red states (*i.e.*, US states whose voters predominantly vote for the Republican Party); and 2.1% lived in US territories (*i.e.*, Puerto Rico and the US Virgin Islands) at the time of their TFMR.

Over a quarter (28.5%) of participants did not have their termination in the state where they lived at the time. Primary reasons for this were TFMR being illegal in their state of residence at the time (16.7%); living close to a state border (*i.e.*, MD and DC; 7.6%); TFMR not being performed in their area (6.9%); trusting the physicians at the facility where the TFMR was performed (6.3%); and undergoing diagnostic workup at the facility where the TFMR was performed (4.2%). Participants reported a mean of 26.32 (over a maximum of 30; *SD* = 3.46) for satisfaction with decision.

See Table 2 for participants' TFMR information.

**Table 2.** Participant Information on Termination for Medical Reasons

	*N* (%)
Region of United States lived at time of TFMR	
West	38 (28.8)
South	29 (22.0)
Midwest	30 (22.7)
Northeast	35 (26.5)
Red/blue State	
Red	43 (29.9)
Blue	98 (68.1)
Other (territories)	3 (2.1)
TFMR performed in state lived	
Yes	95 (72.0)
No	37 (28.0)
No such procedures performed in my area	9 (6.8)
Such procedures illegal in my state	22 (16.7)
I underwent diagnostic workup at this facility	6 (4.5)
I trust the physicians at the facility where the TFMR was performed	7 (5.3)
Other	11 (8.3)

TFMR, termination for medical reasons.

### Stigma and decision satisfaction

In a yes–no question, over half (53.0%) of the participants said their decision had been stigmatized by somebody. In a follow-up question, friends (23.5%) and relatives who were not immediate family (22.0%) were the most commonly reported sources of stigma. Few participants reported experiencing stigma from their physician (3%) or psychologist/psychiatrist (0.8%). See Table 3 for descriptive statistics on stigma experiences.

**Table 3.** Stigma and Social Support Experiences

	*N* (%)
Decision criticized or stigmatized by anybody	
Yes	70 (53.0)
No	62 (47.0)
Stigmatized by	
Partner	0
Immediate family	18 (13.6)
Relative (not immediate family)	29 (22.0)
Friend	31 (23.5)
Physician	4 (3.0)
Psychologist/psychiatrist	1 (0.8)
Other	19 (14.4)
Greatest support	
Partner	120 (90.9)
Immediate family	72 (54.5)
Relative (not immediate family)	12 (9.1)
Friend	66 (50.0)
Physician	51 (38.6)
Psychologist/psychiatrist	30 (22.7)
Other	9 (6.8)

Analysis of variance (ANOVA) results did not reveal significant differences in stigma reporting (yes–no) by demographics, personal political leaning, or religious affiliation. There were no significant differences in stigma reporting (yes–no) by political leaning of the state where participants lived at the time of TFMR. Statistical analyses did not reveal a significant correlation between stigma and decision satisfaction.

Results from *t*-tests did not show mean group differences in decision satisfaction by (1) whether participants had experienced stigma (yes–no) or (2) who had stigmatized their decision. Linear regression by source of stigma was not significant, *p* = 0.66.

### Social support and decision satisfaction

Participants were asked to identify who had offered them the greatest support after making a decision about pregnancy termination. Participants selected the following as their greatest sources of support: partners (90.9%), immediate family members (54.5%), and friends (50%). Some (38.6%) participants said their greatest support after making a decision about pregnancy termination had been their physician and about 20% reported their greatest source of support had been a psychologist or psychiatrist. See Table 3 for descriptive statistics on social support.

*T*-tests showed that decision satisfaction was significantly lower in participants who had one source of support (*M* = 24.47, *SD* = 5.02) than in participants who had more than one source of support (*M* = 26.60, *SD* = 3.06), *t* = 2.527, *p* = 0.01. *T*-test results also showed that participants who received support from a relative had greater decision satisfaction (*M* = 28.17, *SD* = 2.12) than participants who did not receive support from a relative (*M* = 26.11, *SD* = 3.52), *t*(130) = 1.983, *p* = 0.049.

Finally, participants who received support from their physician had greater decision satisfaction (*M* = 27.18, *SD* = 3.04) than participants who did not receive support from a physician (*M* = 25.74, *SD* = 3.62), *t*(130) = 2.357, *p* = 0.02. No other statistically significant group differences were found between those who reported support from other sources (partner, immediate family, psychologist/psychiatrist, friend, and other) and those who did not.

Physician support was the only source that predicted decision satisfaction in a linear regression, *F* (1, 131) = 5.94, *p* = 0.02, *R*^2^ = 0.04.

## Discussion

Our study is novel, in that it focuses on perceptions on abortion stigma, social support, and decision satisfaction of a population that has been understudied, that is, individuals who have had a TFMR,^46^ and deserves urgent attention given the current US political and health care climate around abortion.^1,2^ While the relationship of abortion stigma and social support with mental health outcomes has been explored in the past,^11^ to our knowledge, no research has explored the association of these two constructs (*i.e.*, abortion stigma and social support) with decision satisfaction in individuals who have had a TFMR.

Multiple large studies conducted in the United States have shown that stigma is experienced by the majority of individuals seeking to terminate their pregnancies.^11,12,19,22,24^ Our results show that this is also the case in individuals who have had a TFMR. Interestingly, 47% of our study's participants did not endorse experiencing stigma. In the context of current research demonstrating that experiencing stigma may hinder support-seeking behaviors,^11^ it is possible that our participants, who had joined a support group, may have been more empowered than others who had a TFMR, but did not join such groups.

In addition, the possibility of recall bias exists—participants in our study may recall stigma as less in the context of a supportive group of individuals who have had a similar experience (TFMR). Our study did not find a significant relationship between stigma experiences and satisfaction with the decision to have a TFMR. These findings align with recent research by Kerns et al., who have shown that perceived public condemnation (*i.e.*, stigma) was not linked to psychological outcomes in individuals who undergo TFMR.^11^ As hypothesized by Kerns et al.,^11^ it is possible that having a medical need as the reason for the termination may act as a buffer against higher stigma leading to lower decision satisfaction.

In line with research by Mosley et al.,^47^ in the United States, abortion in the setting of fetal anomalies is connected to higher social acceptability. In addition, it is hypothesized that participants had developed strong internal and external coping resources that helped to mitigate negative outcomes, which aligns with previous research.^48,49^ Social support may also have acted as a buffer against stigma being linked to decision satisfaction.

The majority of participants in this study identified at least one source of support. In line with existing research on individuals who have had a TFMR, we found that participants generally were supported by their loved ones.^39^ Having multiple sources of social support, including partners, immediate family members, and friends, was related to greater decision satisfaction for individuals who have had a TFMR.^46^

While the variance in decision satisfaction explained by provider support is low, the significant main effect between physician support and decision satisfaction further suggests that it is important for individuals who seek a TFMR to have supportive medical care staff throughout their TFMR procedure, as supportive care has the potential to impact decision satisfaction and well-being after the termination.^50^ Health care providers and loved ones can help to ameliorate the distressing impact of a TFMR through learning about and supporting the multifaceted process of ending a wanted pregnancy.^32,46,50–53^

### Limitations

Limitations are present within this study despite its novel conceptualization and methodological strengths. First, participants self-selected into this study on Facebook. While the use of a convenience sample may impact the generalizability of our results, social media platforms are frequently used to recruit participants for health research. The lack of racial, ethnic, and socioeconomic diversity of our sample limits generalizability of results to the broader population of individuals seeking TFMR in the United States. In the future, this could be mitigated by using social media recruitment strategies in conjunction with those that do not require internet access, such as distributing flyers within the community. In addition, internet recruitment could focus exclusively on marginalized communities (*e.g.*, Black, Indigenous, and People of Color individuals who have an abortion).

Second, our study used only self-report measures, for which responses can be affected by recall bias, social desirability, and introspective ability. This is especially the case when questions are on triggering topics such as stigma. Our sample may consist of individuals who hold more sociocultural privilege and safety than others.^18^ Yet, self-reports are commonly used in health research because they provide access to sensitive topics among often marginalized populations such as individuals who have had a TFMR.^38,39^

Third, we opted to include social support and stigma as part of the demographic questionnaire (vs. independent measures) to prevent participant burnout; this likely inhibited the in-depth exploration of subconstructs such as internal versus external stigma or quality of social support. While, generally, research on health-related stigma has been constrained by the lack of validated instruments that distinguish among various stigma-related constructs,^54^ future research could employ a measure developed specifically for abortion such as the Individual Level Abortion Stigma scale.^55^

Fourth, our study was conducted in November/December of 2021, almost immediately before Roe versus Wade was overturned. A scientific understanding of individuals' decision satisfaction and potential predictors (especially social support and stigma) in the post-Dobbs sociopolitical climate is warranted.

Finally, there is potential for reverse causality in our study—that is, low current decision satisfaction may inform perceptions of social support and stigma at the time of termination.^11^ Future research that is longitudinal may provide further insight into how these complex variables are connected.

### Implications for practice and policy

In the context of TFMR, social support is associated with higher rates of post-TFMR decision satisfaction. Health care providers must urge patients to capitalize on their social support through engaging in activities such as finding relevant support groups, especially those organized by other parents who can empathize with their experiences.^23,56^

In addition to demonstrating support for connecting parents who have gone through a TFMR, results from this study are novel, in that they provide a more nuanced understanding, specific to TFMR, of the importance of having multiple social support resources and the important role played, in part, by supportive and informed health care providers. While participants may feel limited in the availability of social support due to factors such as socioeconomic status or geographic residency in the United States, it is important for providers to highlight the possible benefits of support groups and talking with someone who can empathize with their experiences.^23,56^

The intense politicization of pregnancy termination in the United States conceals its status as a health and health care issue.^1^ For people to more confidently, safely, and comfortably discuss their experiences with termination, public policy that supports an individual's autonomy in decision-making must be advanced.^26,57^

Results from the present study may inform training for providers working with clients who seek a TFMR, which should focus on medical competence and also on how to adequately and sensitively support these individuals and families. Considering that the process of terminating a pregnancy could be possibly traumatic for some families and that a post-Dobbs landscape represents a barrier to care for many patients, patients and their partners must have access to providers who can center their needs and respond to their concerns related to abortion in a way that is evidence based, timely, and nonstigmatizing.

Stigmatization is deeply contextual and dynamic^3^; the current sociopolitical climate in the United States has exacerbated the need for providers to strategically manage information about their relationship to abortions. The harassment and violence that providers experience at abortion clinics also contributes to providers' experiences of stigma and burnout.^3,58,59^

Future research can focus on exploring (1) what types of provider support (informational versus compassion) contribute most to decision satisfaction; (2) how patient-perceived stigma specifically related to TFMR impacts patient treatment outcomes, such as satisfaction with physician/medical care provided; and (3) the impact of provider-experienced stigma on patient decision satisfaction and other treatment outcomes.

## Abbreviation Used

TFMR: termination for medical reasons
